# Editorial: Radicalization and deradicalization: Processes and contexts

**DOI:** 10.3389/fpsyg.2022.1059592

**Published:** 2022-10-17

**Authors:** David A. Winter, John F. Morrison, Kees van den Bos

**Affiliations:** ^1^Department of Psychology, Sport, and Geography, University of Hertfordshire, Hatfield, United Kingdom; ^2^School of Law and Criminology, Maynooth University, Maynooth, Ireland; ^3^Department of Psychology and School of Law, Utrecht University, Utrecht, Netherlands

**Keywords:** radicalization, deradicalization, extremism, violence, processes

In a world in which polarization of views is all too apparent, and where extremism is sometimes expressed in violent and terrorist acts, understanding of the factors and processes involved in radicalization, and in the occasional transition from extreme views to violent actions, is of crucial importance. This is also imperative when we want to understand processes of deradicalization and how to counter radicalization into violent extremism. The fostering of such understanding was the principal aim of this Frontiers Research Topic.

Previous work in the field has considered various different psychological and psychosocial aspects of radicalization, drawing upon a range of theoretical perspectives. The continuation of this can be seen across the papers collected in this Research Topic.

A major focus of several of the papers concerns the ‘push, pull, and personal' factors (Vergani et al., 2020) that may predispose an individual to become radicalized. At the personal, individual level, Braddock et al. provide evidence of the role of Machiavellianism (but not other “dark tetrad” personality traits), which interacted with narrative exposure and vividness to amplify the persuasive effect of terrorist narratives. Grimbergen and Fassaert point to the relevance of psychiatric disorders, self-sufficiency problems, and reported adverse childhood experiences, finding high levels of these in people suspected of violent extremism.

Turning to the relationship of the individual to the group, Isenhardt et al. provide evidence that identity diffusion increases approval of left-wing and Islamist extremist attitudes and mediates somewhat the influence of parenting on extremist attitudes. People who have experienced identity diffusion may be particularly vulnerable to identity fusion, for example with an extremist group, and Martel et al. present research findings indicating that identity fusion is a significant predictor of fighting and dying for a cause, as are sacred values and moral convictions, with identity fusion being the strongest predictor of endorsement of self-sacrifice, particularly when the validity of the personal self is under threat. Mason et al. also provide evidence that identity fusion is prominent in political activists, and associated with willingness to undertake extreme behavior; that becoming an activist provides individuals with a clearer and more positive view of themselves, in contrast with extreme negative views of the opposing group; and that similar processes operate in people with contrasting political views.

A special case is made for the relevance of perceived injustice and unfairness. For example, Jansma et al. argue in their theoretical model that perceived unfairness plays a major role in radicalization of people protesting on matters of climate change. Evidence of the association between perceived group-based injustice and support for, and intention to engage in, violent extremism is provided by Rottweiler and Gill, who found this to be particularly so in individuals with high needs for uniqueness and status and less in those high in trait forgiveness, demonstrating strong self-control, or showing critical and open-minded thinking styles. Support for radical action may also be influenced by incidences of such actions, and Schumann et al. provide evidence that the number of attacks on an ingroup was not related to public support for terrorism but number of attacks on an outgroup was. Finally, while some of the papers consider responses to extremist messages, Prentice and Taylor examine the messages themselves, finding considerable overlap between extremist and non-extremist material, which they interpret as being more to do with resistance and positioning than with adoption of an extremist master-narrative by non-extremist authors.

The papers in the Research Topic demonstrate the richness of research on radicalization. In particular, they indicate possibilities for cross-fertilization and theoretical integration, not only within psychological perspectives, but also between these and work in the sociological and historical fields (as shown, for example, in the paper by Jansma et al.) to provide a multi-layered understanding of radicalization in terms of individual and social factors and the influences to which the person is exposed. The empirical papers also demonstrate the utility of a wide range of research methods, including questionnaires, interviews, surveys, repertory grid technique, and textual analysis.

A limitation of research in the area is indicated by the fact that only two of the eight empirical papers in the Research Topic actually focused on participants who may already have been radicalized. While this is a possible weakness of the papers, the study of aspects of radicalization in wider groups of participants does indicate that it is a phenomenon that also involves “normal” psychological processes that, although they may lead to extreme views, do not necessarily result in violence. We would therefore take issue with definitions of radicalization or extremism (e.g., those quoted in the paper by Isenhardt et al.) that seem automatically to equate these with violence or with rejection of particular values, such as those associated with a “Western” worldview. Perhaps the most important implication of viewing radicalization as possibly involving both normal and abnormal psychological processes is that the insights from these analyses may suggest successful and fruitful approaches to deradicalization. Thus, it is our hope that, taken together, the papers collected in this Research Topic may contribute to efforts to prevent violent extremism, in parallel to fostering the continued academic debate.

It is noteworthy, for example, that there are indications that it might be valuable to tailor interventions for specific groups of people: for example, with those who are high in identity fusion with a particular group, interventions directed at diminishing of relational ties to this group coupled with redirection of their beliefs and passions elsewhere (Martel et al.); and with highly Machiavellian people, development of counter-messages that may appeal to them and neutralize the persuasive effects of terrorist propaganda (Braddock et al.). There are also indications of the possible value of involvement of health and social care professionals in programmes countering violent extremism (Grimbergen and Fassaert) and that such programmes should not be purely risk-oriented but should also promote protective factors (Rottweiler and Gill).

In short, we hope that the papers in the Research Topic will stimulate further research in the field in different cultural settings, in particular focusing on the interaction of the psychological, social, and contextual factors involved in radicalization, its escalation to violent extremism, and deradicalization.

## Author contributions

DW, JM, and KvdB conceived, and shared the task of editing, the Research Topic. DW wrote the first draft of the editorial, to which JM and KvdB then contributed. All authors contributed to the article and approved the submitted version.

## Conflict of interest

The authors declare that the research was conducted in the absence of any commercial or financial relationships that could be construed as a potential conflict of interest.

## Publisher's note

All claims expressed in this article are solely those of the authors and do not necessarily represent those of their affiliated organizations, or those of the publisher, the editors and the reviewers. Any product that may be evaluated in this article, or claim that may be made by its manufacturer, is not guaranteed or endorsed by the publisher.
